# Prediction of B-Cell Epitopes in Listeriolysin O, a Cholesterol Dependent Cytolysin Secreted by *Listeria monocytogenes*

**DOI:** 10.1155/2014/871676

**Published:** 2014-01-02

**Authors:** Morris S. Jones, J. Mark Carter

**Affiliations:** Western Regional Research Center, Agricultural Research Service, U.S. Department of Agriculture, 800 Buchanan Street, Albany, CA 94710, USA

## Abstract

*Listeria monocytogenes* is a gram-positive, foodborne bacterium responsible for disease in humans and animals. Listeriolysin O (LLO) is a required virulence factor for the pathogenic effects of *L. monocytogenes*. Bioinformatics revealed conserved putative epitopes of LLO that could be used to develop monoclonal antibodies against LLO. Continuous and discontinuous epitopes were located by using four different B-cell prediction algorithms. Three-dimensional molecular models were generated to more precisely characterize the predicted antigenicity of LLO. Domain 4 was predicted to contain five of eleven continuous epitopes. A large portion of domain 4 was also predicted to comprise discontinuous immunogenic epitopes. Domain 4 of LLO may serve as an immunogen for eliciting monoclonal antibodies that can be used to study the pathogenesis of *L. monocytogenes* as well as develop an inexpensive assay.

## 1. Introduction

**Figure 1:**
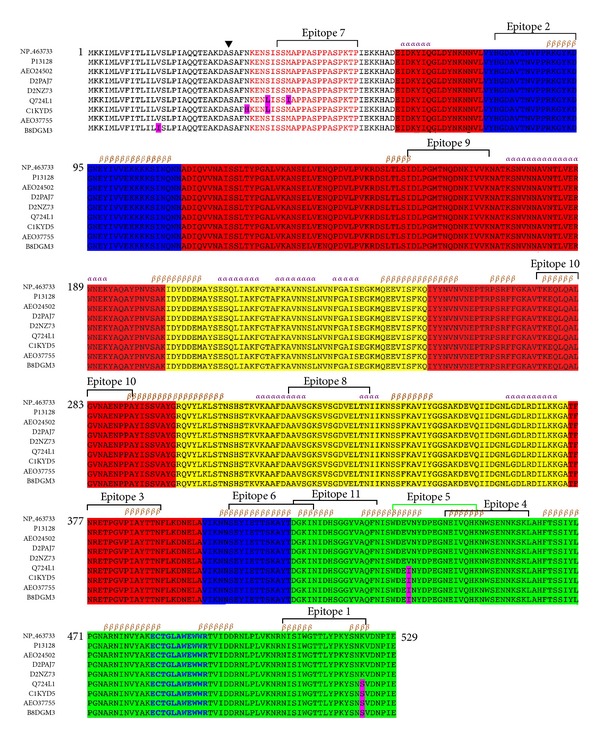
ClustalW alignment of antigenic LLO amino acid sequences from GenBank. Red Letters denote the PEST sequence. Amino acids highlighted in red belong to domain 1, amino acids highlighted in blue belong to domain 2, amino acids highlighted in yellow belong to domain 3, and amino acids highlighted in green belong to domain 4. Amino acids highlighted in magenta are polymorphic. Black triangle denotes the location of signal sequence cleavage site. Purple amino acids denote the location of the undecapeptide sequence, required for the formation of the oligomeric pore complex. The purple *α* and brown *β* symbols above the alignments denote the locations of alpha helices and beta-pleated sheets, respectively, predicted by the Porter Secondary Structure Prediction method.

Listeriosis is a foodborne illness caused by infection with *Listeria monocytogenes*, a gram-positive pathogenic bacterium [1]. *L. monocytogenes *is the only species in the genus *Listeria* that can infect humans [2]. After the bacteria are phagocytosed, listeriolysin O (LLO), an exotoxin, forms a pore in the membrane of the phagosome that allows the bacteria to escape into the cytosol of the phagocyte, where it is adapted to grow [2]. *L. monocytogenes *that is incapable of secreting LLO (Δhly) is not pathogenic [3]. Listeriolysin O is a 529 amino acid protein that is a member of the cholesterol-dependent cytolysin (CDC) family of proteins [4]. LLO is a four-domain poreforming protein that is regulated by pH [5]. LLO also contains a 21 amino acid PEST sequence (Figure 1) in the amino terminus that probably helps to control LLO production in the cytosol [6], as well as an undecapeptide sequence that is important for membrane binding [7] (Figure 1).

Several studies have used bioinformatics to predict the antigenicity of proteins. Frikha-Gargouri et al. used bioinformatics to predict the immunogenicity of the OmcB protein of *Chlamydia trachomatis *[8]. Further experiments confirmed their *in silico *predictions [8]. Jahangiri et al. also used bioinformatics to find a novel region of ActA, a membrane protein found on the surface of *L. monocytogenes* that they predicted would be immunogenic [9]. Another study used bioinformatic screening as well as three-dimensional modeling to show that several regions in the Bap protein, a surface protein found on the surface of *Acinetobacter baumannii*, had a high probability of eliciting antibodies [10].

Monoclonal antibodies (MAb) have many functions. They can be used to study structure/function of a protein, pathogenesis of an organism, and/or quantitative analysis of a protein [11, 12]. To study the pathogenesis of *L. monocytogenes*, it is necessary to map the function of LLO. Currently, only a few MAbs exist against LLO, none of which are commercially available [11–14]. Site-specific antibodies that are able to neutralize LLO may be useful in the study of toxin membrane binding and pathogenicity. They also have the potential to be used in passive immunization to treat listeriosis.

There are two companies that manufacture ELISAs (BioCompare and MyBioSource) to detect LLO (http://www.biocompare.com/pfu/110627/soids/371711/Assay_Kit/listeriolysin_O and http://www.mybiosource.com/datasheet.php?products_id=705426) [15, 16]. In this study we analyzed LLO using bioinformatics to identify putative immunogenic B-cell epitopes for generating antibodies (Abs) against it. This information will be used to design a recombinant protein that will be used to develop an inexpensive yet sensitive assay for the detection of LLO that will be available for the scientific community at large.

## 2. Methods

### 2.1. Sequence Availability and Similarity Search

In this study we used the NCBI GenBank Protein database (http://www.ncbi.nlm.nih.gov/protein/) to acquire the Listeria proteins listeriolysin O (LLO) (NP_463733) and ivanolysin O (P31831) [17]. The accession numbers for other LLO proteins are listed in Figure 1. To determine the best homologs to listeriolysin O (LLO) we performed a blastp search against the Protein Data Bank database (http://www.rcsb.org) at http://www.ncbi.nlm.nih.gov/blast/Blast.cgi. Suilysin (pdb: 3HVN) [18] had the highest amino acid identity to LLO (compared with the other LLO homologs that were crystallized).

### 2.2. Alignments

Pairwise sequence alignments of LLO were generated using the ClustalW2 Multiple Sequence Alignment at http://www.ebi.ac.uk/Tools/msa/clustalw2/ [19]. The purpose was to determine the number of polymorphisms amongst LLO proteins (Figure 1). In addition, we also generated alignments of LLO-homologs to determine the epitope conservancy between the LLO and ivanolysin O. Individual percent identities were calculated using the EMBOSS Needle Pairwise Sequence Alignment at http://www.ebi.ac.uk/Tools/services/web/toolform.ebi?tool=emboss_needle&context=protein [20].

### 2.3. Molecular Modeling

To generate a molecular model of LLO, we used the molecular modeling program I-TASSER http://zhanglab.ccmb.med.umich.edu/I-TASSER/ [21, 22]. We used I-TASSER as the molecular modeling program because it was ranked as the best server for protein structure prediction in recent CASP7, CASP8, and CASP9 experiments [21, 23, 24]. The (Critical Assessment of protein Structure Prediction) CASP is an international competition to assess the best algorithms in the area of 3D protein structure prediction. We chose SLY (pdb: 3HVN) as a template for modeling purposes because it produced a model with the highest C-score. I-TASSER assigns a C-score to each model it generates [21]. The C-score is a confidence score that estimates the quality of each predicted model. A C-score can range from −5 to 2. A C-score closer to 2 indicates a model with high confidence and a model with a C-score closer to −5 signifies low confidence. The C-score for our model was 2. In addition, SLY has the highest amino acid identity to LLO (45.7%) amongst the four crystallized CDCs.

Amino acids 60–525 of LLO were used to predict a three-dimensional model since these residues correspond to amino acids 32–242 and 245–499 in SLY. Molecular models were prepared in different orientations using POLYVIEW 3D (http://polyview.cchmc.org/polyview3d.html) [25].

### 2.4. Prediction of B-Cell Epitopes

Linear B-cell epitopes were chosen with three different algorithms. ABCPred uses a recurrent neural network to predict B-cell epitopes at http://www.imtech.res.in/raghava/abcpred/ABC_submission.html [26]. ABCPred was created by Saha et al. in 2006 to predict B-cell epitopes in an antigen sequence. Saha et al. used 700 B-cell epitopes and 700 non-B-cell epitopes [26]. Moreover, ABCPred is able to predict epitopes with approximately 66% accuracy using the recurrent neural network [26]. ABCPred assigns scores between 1 and 0 to each epitope it predicts. A score that is closer to 1 indicates a high probability of the epitope existing and a score closer to 0 suggests that the amino acid sequence will not become an epitope. We set the amino acid length to 16 mer and the scoring threshold to 0.8. These conditions are similar to what was used by a similar study with ActA [9], a membrane protein of *L. monocytogenes*. COBEPro was developed by Sweredoski and Baldi in 2008 to predict continuous B-cell epitopes [27]. Specifically, COBEPro uses a support vector machine to predict 7 mer peptide fragments within the query amino acid sequence and then calculates an epitopic propensity score for individual residues based on the fragment predictions at http://scratch.proteomics.ics.uci.edu [27]. Fourteen epitope annotated proteins, an HIV data set, as well as a data set from BciPep were used to validate COBEPro [27].

Larsen et al. developed BepiPred in 2006 for the purpose of predicting linear B-cell epitopes [28]. Larsen et al. used 14 epitope annotated proteins as well as an HIV data set. BepiPred employs the hidden Markov model and a propensity scale method at http://www.cbs.dtu.dk/services/BepiPred/ [28]. We used 0.35, because it is the point at which sensitivity/specificity is maximized in BepiPred. BepiPred analyzes each amino acid independently and does not have a minimum or maximum number of amino acids to predict an epitope. Overlapping epitopes chosen by the three B-cell prediction algorithms were chosen as potential B-cell epitopes.

Discontinuous epitopes were predicted using ElliPro Antibody Epitope Prediction at http://tools.immuneepitope.org/tools/ElliPro/iedb_input [29]. ElliPro, when compared to six other software programs that predict discontinuous epitopes, was determined as the best algorithm for predicting discontinuous epitopes inferred from 3D structures [29]. ElliPro predicted three-dimensional discontinuous epitopes on the surface of LLO based on the molecular model described above. ElliPro uses three algorithms to predict discontinuous epitopes. It approximates the protein shape as an ellipsoid, calculates the residue protrusion index (PI), and clusters the neighboring residues based on their PI values. ElliPro generates a PI score (PI) for each predicted epitope. Our cutoff for PI scores was 0.745 (compared to the default value of 0.8), which produced results that generally agreed with BepiPred.

### 2.5. Immunoinformatic Analysis

Important properties for predicting B-cell epitopes are flexibility, hydrophilicity, and linear epitope predictions. We analyzed the linear epitope predictions, flexibility, and hydrophilicity of LLO using the BepiPred linear epitope prediction [28], Karplus and Schulz flexibility prediction [30], and Parker et al. hydrophilicity prediction [31] algorithms, respectively, at http://tools.immuneepitope.org/tools/bcell/iedb_input. A similar tool that we did not employ here, Bcepred (http://www.imtech.res.in/raghava/bcepred/bcepred_submission.html) also uses physicochemical properties to predict B-cell epitopes similar to BepiPred [32].

## 3. Results

### 3.1. Sequence Conservation of LLO

An alignment of all completely sequenced amino acid coding sequences of LLO from several *Listeria* species revealed that it is highly conserved with only six polymorphic sites (Figure 1). However, only five of those are present in the mature protein (Figure 1). We used NP_463733 as a reference amino acid sequence. A similarity search revealed that the amino acid sequence of LLO was 81.7% and 79.6% identical to LLO (*L. seeligeri*) and ivanolysin O (*L. ivanovii*), respectively. In contrast, NCBI-BLASTn searches against Listeria species *L. grayi*, *L. innocua*, *L. marthii *genomes (members of the genus Listeria that have their genomes sequenced) yielded no LLO homologs. The genomes of species *L. fleischmannii *and *L. rocourtiae *have not been sequenced.

### 3.2. Three-Dimensional Prediction of Listeriolysin O

**Figure 2:**
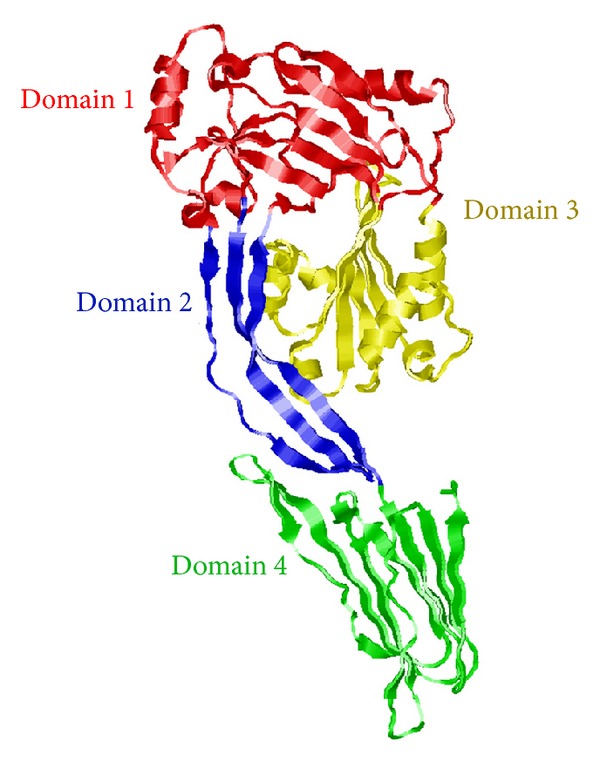
Three-dimensional molecular ribbon model of LLO toxin based on the crystal structure of suilysin (SLY). Red ribbons denote amino acids highlighted in domain 1, blue ribbons denote amino acids highlighted in domain 2, yellow ribbons denote amino acids highlighted in domain 3, and green ribbons denote amino acids highlighted in domain 4.

Since there is no LLO crystal structure, we created a molecular model of LLO to visualize the locations of the predicted B-cell epitopes (Figure 2). The SLY structure was chosen as a template to model LLO, since it has high amino acid identity to LLO (45.7%) amongst crystallized LLO homologs. Comparison of the SLY, perfringolysin O (PFO), intermedilysin (ILY), and alveolysin (ALO) crystal structures in the CDC protein family demonstrated that the aforementioned proteins share a similar 3D structure [4].

### 3.3. Immunoinformatic Analysis of 3D Listeriolysin O

**Figure 3:**
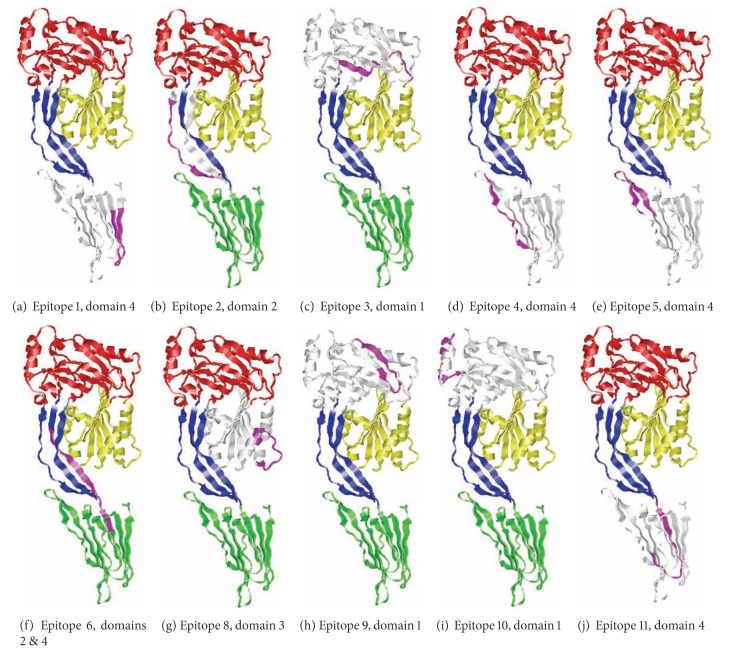
Three-dimensional representation of predicted linear B-cell epitopes. Magenta ribbons designate the position of the predicted linear epitopes. Location of predicted epitope is listed below the epitope number.

**Figure 4:**
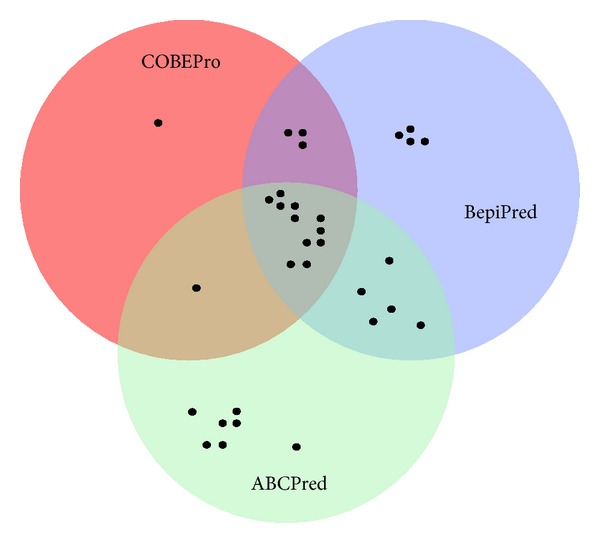
Venn diagram of epitopes detected by ABCPred, BepiPred, and COBEPro. Black dots represent epitopes detected by each B-cell prediction algorithm. Epitopes predicted via ABCPred, BepiPred, and COBEPro are in green, purple, and red circles, respectively. The 11 epitopes that were detected by all three algorithms are in the center of the Venn diagram.

We used amino acids 60–525 of LLO to predict a three-dimensional model. In contrast, we used amino acids 28–529 to analyze the immunogenicity of LLO since the latter amino acids comprise the full-length mature protein [33]. Three different epitope prediction software programs (ABCPred, BepiPred, and COBEPro) were utilized to predict the most immunogenic linear B-cell epitopes on the surface of LLO (Section 2). ABCPred and BepiPred predicted 24 and 18 different potentially immunogenic regions within LLO, respectively, sixteen of which overlapped (Figure 3). Epitopes that did not overlap were not considered for analysis. COBEPro, a B-cell epitope prediction program that we used in conjunction with ABCPred and BepiPred, only recognized 11 of 16 epitopes that were mutually predicted via ABCPred and BepiPred (Figure 3). Four of 11 epitopes that the three software programs agreed upon were located in domain 4 (Figure 4) and one of them overlapped domains 2 and 4 (Figure 4(f)). Three, one, and one epitopes were located in domains 1, 2, and 3, respectively (Figures 1, 3, and 4). In addition, one epitope was predicted to be in the PEST sequence (Table 1 and Figure 1).

**Figure 5:**
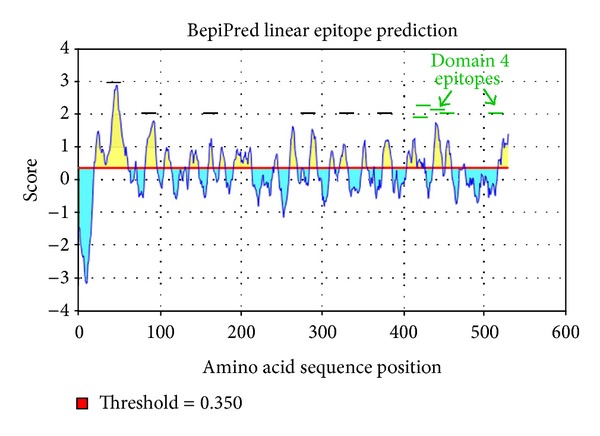
Graphical result of BepiPred prediction for linear B-cell epitopes. Yellow color denotes positive score for linear B-cell epitopes. Linear black lines denote the approximate position of the 11 predicted linear epitopes. Linear green lines denote the approximate position of the predicted linear epitopes in domain 4.

We also evaluated discontinuous epitopes in the LLO molecular model that we created. ElliPro predicted three discontinuous epitopes with PI scores higher than our cutoff: two in domain 4 and one in domain 1 (Figure 5). Discontinuous epitope number 1, located in domain 4, was predicted to touch several residues (Figure 5(a)).

### 3.4. Specificity of Predicted Immunogenic Epitopes

In terms of antibody recognition, changing one amino acid in an antibody epitope can dramatically decrease the antigen-antibody interaction [34]. Four of the 11 predicted epitopes have greater than 80% amino acid identity to ivanolysin O (Table 2). Of the four potentially antigenic B-cell epitopes with high amino acid identity to ivanolysin O, two of them (epitopes 6 and 10) only differ by one amino acid (Table 2). None of the predicted antigenic epitopes were identical to ivanolysin O amino acid sequences, the closest homolog of LLO.

### 3.5. Epitopes in LLO Exhibit Immunogenic Properties

Immunogenic epitopes are accessible on the protein surface, located in flexible regions, and often overlap [35]. Our predicted linear immunogenic epitopes of LLO are located in protein regions that are predicted to be accessible (Figures 4 and 5) and flexible (Figure 7). In addition, two different sets of epitopes overlap in domain 4 (Figures 1 and 5), implying that domain 4 may be immunodominant.

## 4. Discussion

**Figure 6:**
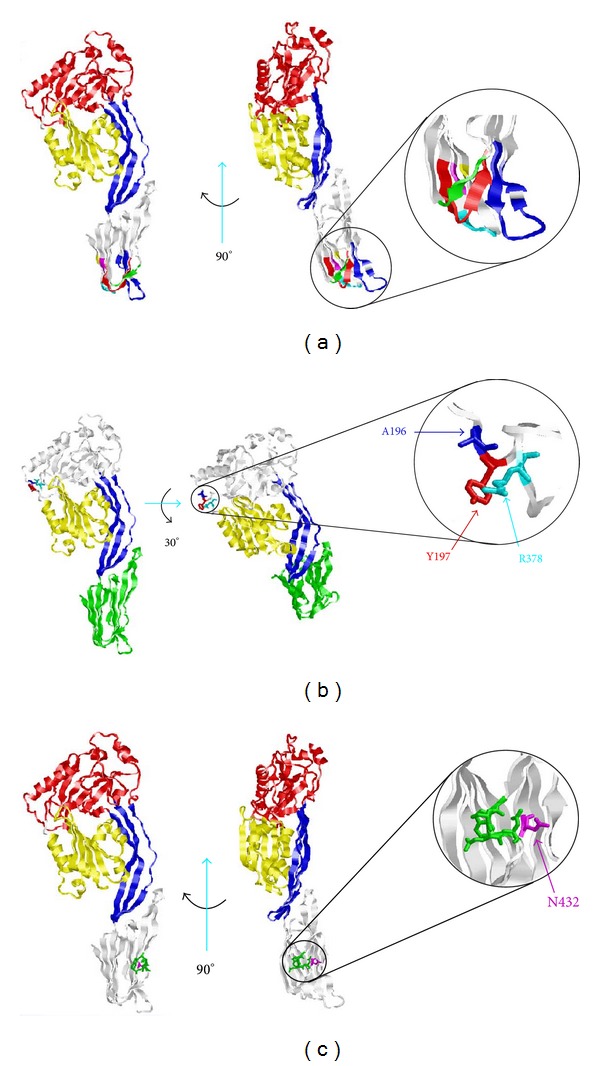
Three-dimensional representation of predicted discontinuous B-cell epitopes. (a) LLO with orientation rotated 90° to the left and enlarged to highlight discontinuous epitope in domain 4 representing amino acids 422–431 (red), 458–462 (green), 480–492 (blue), 494–496 (blue), 510-511 (magenta), 513–516 (aqua), and 521 (yellow); (b) LLO with orientation rotated 30° towards the bottom and enlarged to highlight discontinuous epitope in domain 1 representing amino acids A196 (blue), Y197 (red), and R378 (aqua); (C) LLO with orientation rotated 90° to the left and enlarged to highlight discontinuous epitope in domain 4 representing amino acids N432 (in magenta), and 453–456 (green).

**Figure 7:**
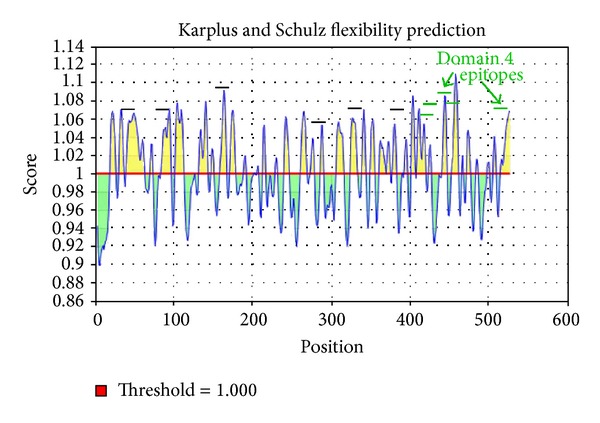
Graphical result of the Karplus and Schulz flexibility prediction for linear B-cell epitopes in LLO. Yellow peaks denote positive score for flexibility throughout the primary amino acid sequence of LLO B-cell epitopes. Linear black lines denote the approximate position of the 11 linear epitopes predicted via BepiPred, ABCPred, and COBEPro. Linear green lines denote the approximate position of the predicted linear epitopes in domain 4.

**Figure 8:**
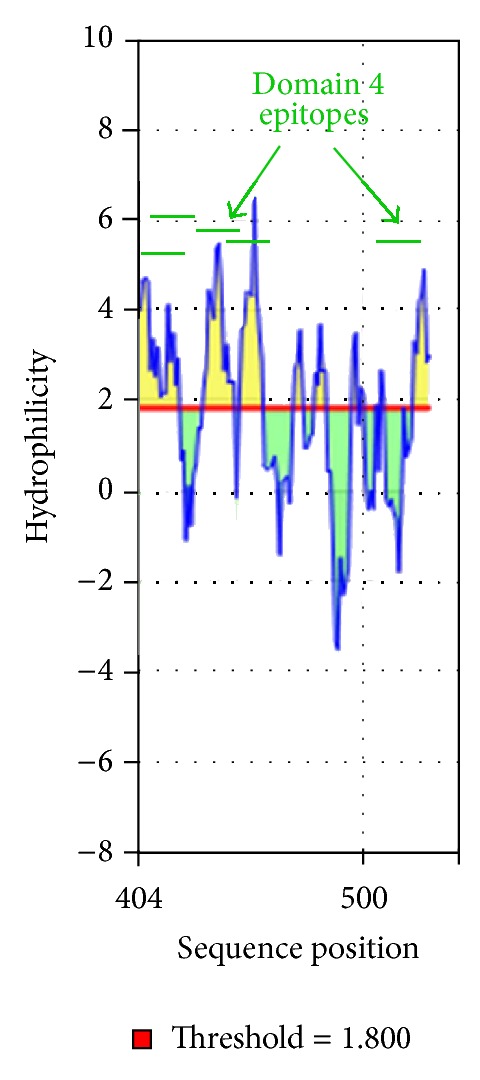
Graphical result of Parker hydrophilicity prediction for linear B-cell epitopes in domain 4 of LLO.

In some instances, when a protein has multiple overlapping B-cell epitopes it is referred to as immunodominant [35]. We predicted 11 antibody epitopes in LLO. Specifically, domain 4 had five of the 11 predicted antibody epitopes, in LLO. Two sets of epitopes were predicted to overlap (Figures 1 and 6). These data imply that domain 4 may be immunodominant. Based on the predicted three-dimensional structure of LLO (Figure 2), this is highly plausible as the linear epitopes in domain 4 are predicted to be accessible and hydrophilic (Figures 4 and 8).

Bioinformatics has been used for many purposes, such as vaccine design, characterization of novel genes, and the discovery of novel viruses [36–39]. Recently, Jahangiri et al. used bioinformatics to predict B-cell epitopes in the ActA protein [9]. Jahangiri et al. used bioinformatic methods similar to our study and found unique sequences in ActA that they plan to use as an antigen to elicit antibodies for a diagnostic test [9]. Bioinformatics has also been used successfully to predict antibody-binding sites for known antibodies [40]. Recently a B-cell epitope prediction software successfully predicted 31 of 32 antigenic sites that were known to bind to antibodies, for an accuracy of 96.88% [40]. The aforementioned studies demonstrate the usefulness of bioinformatics for epitope prediction.

Several characteristics make domain 4 an attractive immunogenic candidate. Domain 4 is the only continuous domain in LLO (Figure 1). Domain 4 also contains more amino acids predicted to be antigenic than the other domains. Furthermore, domain 4 is predicted to be a stable, soluble fragment that does not make significant contact with domains 1–3. The aforementioned point was illustrated for four other crystallized LLO homologs [4].

Bioinformatics of LLO demonstrates that its closest homologs are the LLO protein of *L. seeligeri *and ivanolysin O expressed by *L. ivanovii*. However, *L. seeligeri *does not infect humans, and *L. ivanovii *is not pathogenic [41]. Therefore, even though there is a low probability that an LLO specific monoclonal antibody may have an affinity to ivanolysin O or LLO from *L. seeligeri*, it is unlikely that the latter toxins would be present in contaminated food products consumed by humans. Thus, even if they readily cross-react with LLO *L. seeligeri *and ivanolysin O, antibodies capable of detecting LLO in an ELISA would not be likely to produce a false positive in food screening or clinical tests. Previous molecular models of LLO used PFO as a template [7, 42]. Our LLO molecular model was based on the crystal structure of SLY. This is because SLY has the highest amino acid identity to LLO (45.7%) amongst SLY, PFO, ILY, and ALO—the homologs of LLO for which crystal structures are available. Interestingly, comparison of the SLY, PFO, ILY, and ALO crystal structures in the CDC protein family demonstrated that they share a similar 3D structure [4]. Taken together this implies that our molecular model may be similar to the actual structure of native LLO.

## 5. Conclusions

Bioinformatics of LLO predicted that most of the epitopes deemed likely to be immunogenic were located in domain 4. Furthermore, since it is the only domain in LLO that is continuous, we believe that it has a high probability of eliciting antibodies that could be used to study the pathogenesis of *L. monocytogenes *as well as develop a diagnostic test for LLO detection. This analysis is important because it is focusing our antibody development efforts on domain 4 as an immunogen.

## Figures and Tables

**Table 1:** Potential linear B-cell epitopes in LLO predicted by both ABCpred and BepiPred.

Number	Sequence	Domain	Start position	End position	Score
1	NISIWGTTLYPKYSNK	4	508	523	0.94
2	HGDAVTNVPPRKGYKD	2	79	94	0.93
3	TFNRETPGVPIAYTTN	2	375	390	0.91
4	NEIVQHKNWSENNKSK	4	445	460	0.90
5	WDEVNYDPEGNEIVQH	4	435	450	0.88
6	SEYIETTSKAYTDGKI	4	404	419	0.86
7	SSMAPPASPPASPKTP	PEST	37	52	0.86
8	AAVSGKSVSGDVELTN	3	321	336	0.86
9	IDLPGMTNQDNKIVVK	1	156	171	0.86
10	TKEQLQALGVNAENPP	3	275	290	0.83
11	DGKINIDHSGGYVAQF	4	416	431	0.82

**Table 2:** Comparison of epitope conservancy between LLO (*L. monocytogenes*) and ivanolysin O (*L. ivanovii*). All epitopes were 16 amino acids in length.

Number	Epitope sequence	Amino acid identity of LLO and ivanolysin (%)
1	NISIWGTTLYPKYSNK	68.8
2	HGDAVTNVPPRKGYKD	62.5
3	TFNRETPGVPIAYTTN	68.8
4	NEIVQHKNWSENNKSK	68.8
5	WDEVNYDPEGNEIVQH	68.8
6	SEYIETTSKAYTDGKI	93.8
7	SSMAPPASPPASPKTP	87.5
8	AAVSGKSVSGDVELTN	62.5
9	IDLPGMTNQDNKIVVK	75
10	TKEQLQALGVNAENPP	93.8
11	DGKINIDHSGGYVAQF	81.3
